# Corrupting the DNA damage response: a critical role for Rad52 in tumor cell survival

**DOI:** 10.18632/aging.101263

**Published:** 2017-07-15

**Authors:** Rachel Lieberman, Ming You

**Affiliations:** ^1^ Cancer Center, Medical College of Wisconsin, Milwaukee, WI 53226, USA; ^2^ Department of Pharmacology and Toxicology, Medical College of Wisconsin, Milwaukee, WI 53226, USA

**Keywords:** Rad52, DNA damage response, squamous cell carcinoma of the lung, pre-clinical model, tumor growth

## Abstract

The DNA damage response enables cells to survive, maintain genome integrity, and to safeguard the transmission of high-fidelity genetic information. Upon sensing DNA damage, cells respond by activating this multifaceted DNA damage response leading to restoration of the cell, senescence, programmed cell death, or genomic instability if the cell survives without proper repair. However, unlike normal cells, cancer cells maintain a marked level of genomic instability. Because of this enhanced propensity to accumulate DNA damage, tumor cells rely on homologous recombination repair as a means of protection from the lethal effect of both spontaneous and therapy-induced double-strand breaks (DSBs) in DNA. Thus, modulation of DNA repair pathways have important consequences for genomic instability within tumor cell biology and viability maintenance under high genotoxic stress. Efforts are underway to manipulate specific components of the DNA damage response in order to selectively induce tumor cell death by augmenting genomic instability past a viable threshold. New evidence suggests that RAD52, a component of the homologous recombination pathway, is important for the maintenance of tumor genome integrity. This review highlights recent reports indicating that reducing homologous recombination through inhibition of RAD52 may represent an important focus for cancer therapy and the specific efforts that are already demonstrating potential.

## DNA DAMAGE RESPONSE

DNA damage is a normal occurrence, with the average cell facing up to one million DNA damaging events each day [1]. Defined as a change in the basic structure of DNA, damage includes chemical additions or disruptions to a base of DNA such as crosslinking and DNA adducts or a break in the individual strands of DNA [2]. Consequently, when DNA carrying a damaged base is replicated, an incorrect base may be inserted opposite the site of the damaged base in the complementary strand, leading to a mutation in the next round of replication [3, 4]. DNA damage also occurs when DNA double-strand breaks are restored by an inaccurate repair process, leading to mutations.

Mutations, however, can be avoided if DNA repair systems recognize DNA damage as irregular, and repair the damage prior to replication.

DNA becomes damaged in the course of normal replication, as a result of internal cellular respiration, and also through exogenous means. Endogenously, the human body produces reactive oxygen species (ROS) and internal metabolites which alter DNA [5]. Exogenously, cells face environmental carcinogens, as well as genotoxic and proinflammatory drugs as a part of daily routine [6]. In order to combat these sources of DNA damage, what has evolved is a multifaceted DNA damage response (DDR). This response developed in order to address the need for repair and to activate cell cycle checkpoints to allow for cell maintenance. However, if the repair process is inefficient or non-functional, cumulative DNA damage may result in genetic mutations and chromosomal damage, con-tributing to genomic instability or cancer [7, 8]. Genomic instability signifies an increased tendency of a cell to acquire alterations in the genome during its life cycle [9]. This can be a major driving force for tumori-genesis but can also lead to the demise of a tumor cell if the extent of damage exceeds a sustainable level. In order to preserve genomic integrity, DNA must be protected from such damage caused by exogenous means or generated spontaneously by formation of ROS during normal DNA metabolism [4, 8]. Thus, prompt repair of DNA damage is a crucial protective mechanism in safeguarding the integrity of the cell [4, 10–13].

### DNA damage and genetic risk

Inherent DNA damage due to endogenous mechanisms or unavoidable low doses of environmental carcinogens has a cumulative effect, leaving a mutational signature on the developing cancer genome [14, 15]. The extent of endogenous DNA damage can thus be prognostic for chemoprevention efficacy, one's risk of developing cancer and other genetic diseases arising from *de novo* mutations. For instance, while 80%-90% of lung cancer patients smoke, only a fraction of smokers, (10%-20%) will develop lung cancer in their lifetime [16]. Accordingly, a large population may be exposed to an environmental toxin, but only those who are susceptible to the toxin's effects due to inherent genetic aberrations have an increased risk of developing disease. Therefore, although carcinogens undoubtedly increase cancer risk, other factors such as variation in the DDR likely alter susceptibility to and prognosis in disease.

### DNA damage and cancer

DNA damage occurs in most types of cells including replicating cells, proliferative cells, and in differentiated, non-dividing cells such as neurons in the brain. Cancers, however, occur primarily in pro-liferative tissues when a genetic variant leads to uncontrolled cell growth. Cancerous cells also tend to have high levels of DNA damage and therefore exhibit a heightened level of genomic instability. Thus, the DNA damage response would fundamentally appear to protect the cell from tumorigenesis, and in some cases it does. For instance, mutations in tumor suppressor and DNA damage response genes BRCA1/2 leaves the cellular protein depleted or inactive and increases one's risk of breast and ovarian cancer. However, the DNA damage response functions differently in cancer cells than it does in normal cells, making it a viable anti-cancer target [12].

While it makes sense that an alteration in the DDR would elicit a higher predisposition to develop cancer, conversely looking at the DDR as an actual pro-oncogenic mechanism is not as straight forward. As described above, many cancers will be lacking a portion of or an entire DDR pathway. However, unlike normal cells, cancers will also be associated with replication stress factors such as cell-cycle checkpoint loss, increased transcription, higher levels of metabolic stress, increased ROS formation, and activation of oncogenic drivers [12]. This combination of increased DNA damage and decreased traditional repair capacity leads the cancer cells to become exceedingly more dependent on alternate cellular pathways, leading to vulnerability and instability [10]. In fact, a principal hallmark of cancers is their propensity to accumulate DNA damage and their subsequent genomic instability. Routine treatment of cancer by therapies such as radiation and DNA-damaging chemotherapy target genomically unstable cancer cells based on this standard [12, 17, 18].

### RAD52 and the DDR

Unrepaired or incorrectly repaired DSBs can result in cell death, senescence, or chromosomal aberrations such translocations, deletions and insertions which may induce a loss of heterozygosity [19]. As described above, such chromosomal aberrations are associated with genomic instability and may lead to carcino-genesis. Therefore, cells require on duty mechanisms to quickly repair DSBs. There are two types of repair which address double-strand breaks in DNA: homologous recombination (HR), which uses the sister chromosome as a source of information, and non-homologous end-joining (NHEJ), which does not rely on another source of information [20]. RAD52, a protein important for DNA double-strand break repair in homologous recombination, the more widely used mechanism for eukaryotes. RAD52 mediates RAD51 function in homologous recombinational repair (HRR) in both yeast Saccharomyces cerevisiae and in mammalian cells of mice and humans. Recombination refers to the exchange of nucleotide sequences, aka information, between DNA molecules [21]. HR specifically involves the exchange of DNA between sequences of essentially perfect homology. This process of homologous recombination plays both a crucial role in the mitotic and meiotic cell cycles of most eukaryotic organisms, as well as being the primary mechanism in mitotic cells for repair of double-strand breaks that form due to damage [21]. HR repairs insults of DNA damage such as DNA gaps, adducts, double-strand breaks, and interstrand cross-links and is mediated by a group of conserved genes of the Rad52 epistasis group [21, 22]. The Rad52 epistasis group earned its title name when Rad52 was initially studied in *S. cerevisiae*, and named a key player in recombinational repair [23]. The RAD52 epistasis genes (RAD50, RAD51, RAD52, RAD54, RAD55, RAD57, RAD59, MRE11, and XRS2) are mainly RAD genes involved in recombinational repair of DSBs [21, 24–26].

RAD52, of the Rad52 epistasis group, functions similarly to the well-known tumor suppressor protein BRCA2 in providing high-fidelity replication of DNA through the aforementioned HR pathway [27]. Hence, RAD52 appears to work alongside the BRCA2 pathway as part of redundant repair mechanisms. In fact, RAD52-RAD51 foci, whether ionizing radiation-induced or S phase-associated, form equally well in the presence or absence of BRCA2. Such studies confirm RAD52 to be part of an independent and alternative repair pathway of homologous recombination that can respond to DNA double-strand breaks and replication stalling independently of BRCA2 [27, 28]. However, despite its similarities to the renowned breast cancer tumor suppressor and its hypothesized purpose of repairing damaged DNA in order to maintain viability in normal cells, differential expression of the RAD52 gene has been shown to function in the progression of tumorigenesis both on its own accord, as well as in the absence of BRCA proteins [27, 29–34].

## PRO-ONCOGENIC NATURE OF RAD52

### Association of RAD52 SNPs with cancer

Advances in genomics including DNA sequencing technology and bioinformatics have allowed us to identify some of the molecular characteristics that define different cancers. These include both novel characterizations of genes found to be involved in tumorigenesis, as well as genetic mutations in oncogenes and tumor suppressors that effect tumor growth [35]. Analyses of patients have revealed distinct differences in the tumor genomes of comparative populations such as smokers and non-smokers or between cancer patients and the normal population [36]. Such studies which use comprehensive genomic analysis led the way for the application of mutation/genetic driver-targeted therapy, based not only on organ site but rather on cellular pathway and gene or protein modifications.

Recent evidence has begun to show that genetic variants also play a role in cancer progression and prognosis in patients [37, 38]. Already shown to work with proteins such as RAD51 and OGG1 to repair DNA damage and increase cellular resistance to oxidative stress, RAD52 was first associated with NSCLC risk through a genome-wide association study conducted in Europeans which linked the 12p13.33 locus containing *RAD52* with squamous cell lung cancer risk [39, 40]. This preliminary study designated variation in *RAD52* as a risk factor for squamous cell lung cancer by comparing 5,355 European ever-smoker lung cancer patients with 4,344 smoking control subjects. Squamous cell lung cancer (LUSC) development is strongly associated with smoking, thus, it made sense that there would be an increased need for DNA repair machinery due to the genotoxic effects of long-term tobacco use [41].

The Pardini group demonstrated the role of single nucleotide polymorphisms (SNPs) residing in miRNA target sites of DSB repair genes and their association with colorectal cancer (CRC) risk and clinical outcome [42]. Both MRE11A and RAD52 share miRNAs predicted to bind to regions in their DNA where SNPs have been associated with survival [42]. Further investigation into the miRNAs predicted to bind to both RAD52 and MRE11A at the location of the aforementioned SNPs determined that many of the selected miRNAs are expressed in colon tissue. Among the data, two miRNAs (miR-1296 and miR-296–5p), predicted to bind both genes were shown to be dysregulated in cancer and other diseases. In particular, miR-296–5p has frequently been associated with cancer prognosis [43, 44]. Thus, recalling how coordination between genes involved in DNA repair pathways is fundamental for maintaining genome stability, this study suggests the importance of post-transcriptional gene regulation by miRNAs. As gene expression modulators, microRNAs may be able to regulate DNA repair through SNPs in miRNA binding sites on DNA repair genes. These differences may be one distinct factor affecting microRNA binding, protein expression, and could ultimately account for individual differences in the DNA repair capacity [45].

Alterations in DNA repair is also at the forefront of cervical cancer, especially in overcoming therapeutic resistance. A study by Shi et al. evaluates three potentially functional RAD52 SNPs and their association with *in vitro* platinum resistance and prognosis in cervical squamous cell carcinoma (CSCC) patients [46]. Platinum-based chemotherapy is a common treatment regimen in cervical squamous cell carcinoma (CSCC), as well as other cancers [47]. A major problem that accompanies this type of treatment is the occurrence of primary or acquired drug resistance where the DNA repair response works extensively to repair or remove platinum-based DNA adducts that would otherwise lead to cell death [48]. This group found two SNPs that correlated with both RAD52 protein expression and either carboplatin or nedaplatin resistance. Progression-free survival was significantly lower in patients with at least one variant allele [46]. Thus, in correlating variants in DDR genes with protein expression and subsequent survival rates, variants in DDR genes such as *RAD52* may be able to predict prognosis in cervical cancer patients.

In accordance with the previous studies, we evaluated the concept of DNA repair modulation in lung tumorigenesis in further examining the role of *RAD52* [34]. We discovered two cis-expression quantitative trait loci (eQTL) SNPs in the *RAD52* gene that are associated with its expression, as well as with LUSC risk. Subsequently, we showed that amplification of the genomic region 12p13.33, which contains *RAD52*, was associated with the development of LUSC and that somatic overexpression of *RAD52* was significant in LUSC tumors from our own patient cohort [34]. The aforementioned information regarding altered expression of *RAD52* suggested a corroborative change in functionality. These genetic observations, thus, guided our group to functionally demonstrate that over-expression of *RAD52* in lung cancer cell lines led to an increased rate of cell proliferation. Conversely, depletion of *RAD52* not only decreased the rate of tumor cell growth but also increased DNA damage and subsequently halted tumor cells at the G2 phase of the cell cycle [34]. This data aligns with that of others in connecting variation in *RAD52* expression to the survival of tumor cells from numerous organ sites. Such identification and understanding of susceptibility loci will likely play a valuable role in personalized treatment therapies for patients now and in the future.

### *RAD52* expression impacts tumorigenesis

Although RAD52 functions in a similar manner to the BRCA2 tumor suppressor, repairing DNA damage in order to stabilize cell homeostasis and viability, expression of RAD52 has recently been linked to several different forms of tumorigenesis (Table 1). One of the first studies linking RAD52 to tumorigenesis was through oxidative DNA damage and genetic instability in hepatocarcinogenesis [49]. This study examined whether defects in DNA repair pathways contribute to the acceleration of liver cancer in TGFalpha/c-myc mice. Starting on a broad scale, a cDNA expression array containing 140 known genes and multiplex RT-PCR was used to compare the basal expression levels of DNA repair genes at the dysplastic stage. Surprisingly, Rad52 was found to be upregulated in 10-week-old TGFalpha/c-myc and c-myc transgenic livers, respectively, compared with wildtype controls. These data became some of the first to suggest that expression of DNA damage response factors may directly contribute to acceleration of cancer [49].

**Table 1:** Pro-Oncogenic Roles of Rad52

**Breast/Ovarian**	Upon restoration of expression, levels of miR-302a, a breast cancer radiotherapy sensitizer, inversely correlated with RAD52	[80]
	RAD52 as a therapeutic target against familial breast and ovarian cancer with defective BRCA1/2/PALB2 genes	[32]
	Prevention of brain metastasis of triple-negative breast cancer through Vorinostat's targeted down-regulation of Rad52	[65]
**Liver**	Association between RAD52 SNPs and HBV-related HCC risk	[81]
	Rad52 up-regulated in TGFalpha/c-myc and c-myc transgenic mouse livers	[49]
**Lung**	Susceptibility locus identified for squamous cell lung carcinoma at 12p13.33 (RAD52)	[39]
	Significant amplification of 12p13.33 and somatic overexpression of RAD52 in LUSC tumors; functional studies link Rad52 to tumorigenesis	[34]
**Leukemia**	RAD52 F79 aptamer found to induce synthetic lethality in BRCA1- and/or BRCA2-deficient leukemia	[30]
	6-OH-dopa inhibits RAD52 ssDNA binding domain and proliferation of BRCA-deficient leukemia cells [80, 81, 82, 83]	[83]
**Lymphoma**	Genetic deletion of Rad52 in ATM knockout mice reduced HR and development of T-cell lymphomas	[33]
**Cervical**	RAD52 variants and protein expression can predict platinum resistance and possibly prognosis in cervical cancer patients	[46]
**Colorectal**	miRSNPs in Rad52 are involved in the maintenance of genomic stability and may affect CRC susceptibility and prognosis	[42]

Shortly after RAD52 was found to be upregulated in liver cancer, a study by the Barlow group connected expression of the murine Rad52 gene with the disease Ataxia Telangiectasia (A-T). Cancer predisposition caused by A-T likely results from the loss of ATM kinase activity and excessive homologous recombination, leading to tumors with aberrant levels of DNA amplifications, deletions, and translocations [50]. This group found that knockout of the Rad52 gene in an *in vivo* ATM−/− mouse model led to not only an increased latency period to develop T-cell lymphoma, but also an increased lifespan and decreased overall tumor incidence in Rad52 knockout mice compared to Rad52 wildtype [33]. This newfound ability to decrease tumorigenesis and increase survival by depletion of Rad52 in a tumorigenic environment was hypothesized to stem from a reduction in excessive intrachromosomal recombination found in the absence of ATM [33].

RAD52 expression was linked to loss of a BRCA gene in tumorigenesis when the Skorski group published its impact on BCR-ABL1-mediated leukemogenesis [30]. As mentioned above, DNA repair mechanisms are often times altered in malignant cells to accommodate the heightened level of genotoxic stress [51]. BCR-ABL1- mediated leukemia, which is deficient in BRCA-controlled DNA repair, specifically relies on RAD52 to repair increased levels of damage in the leukemia stem cell environment due to enhanced ROS-induced damage [30]. Thus, by inhibiting the binding of RAD52 to DNA through targeting one of its two DNA binding domains using a small peptide aptamer, a deadly level of DSBs accumulate in malignant leukemia cells. Consequently, hindering the binding capability of RAD52 prohibited the proliferative and clonogenic potential of CML-CP leukemia stem and progenitor cells due to the cells’ oncogenic addiction to Rad52 DNA repair. Since the concept of synthetic lethality relies on the addiction of cancer cells to a single DNA repair pathway, whereas normal cells operate their DNA damage response by way of two or more mechanisms, non-cancerous cells did not have to rely on Rad52 for constant repair and were left unharmed [30, 52].

Concomitantly, unpublished data from our group suggests Rad52^−/−^ mice are more resistant to squamous cell lung carcinoma compared to wildtype [84]. Wildtype or Rad52^−/−^ mice were randomized into two groups and treated topically with 0.04 M NTCU twice a week for 40 weeks. Lungs were fixed and stained with H&E and examined histologically to establish the types of lesions (invasive SCC, SCC *in situ*, or bronchial hyperplasia/metaplasia), as well as markers of LUSC and proliferation [84]. Our novel observations suggest that knockout of the Rad52 gene in an *in vivo* mouse model of carcinogenesis decreases murine lung hyper-plasia, *in situ* carcinoma and lung SCC [84]. These observations, complementary to genetic findings, further strengthen the notion of *RAD52* as a potential oncogene and implicates a major role for the process of re-combinational repair in determining risk for LUSC [84].

## THE DDR AS A MECHANISM IN TUMOR PROGRESSION

### Maintenance of genomic stability

Cancer is the result of uncontrolled cell proliferation, generally due to unresolved DNA mutations which allow for the cell cycle to bypass any warnings or red flags that would normally cause the replication process to halt. It is also understood that the genome accrues genomic alterations during normal cell division and tissue growth and that this, coupled with alterations in DNA repair, can be a driving force for said tumori-genesis [9]. However, although the DNA damage response has been undoubtedly linked to cancer at every stage of progression, the exact mechanisms underlying the DDR at different stages of tumorigenesis are not fully understood. Evidence demonstrates the bipartisan role that the DDR plays in maintaining genomic stability for both the viability of normal, as well as cancer cells.

Unlike in a healthy system, the biological environment surrounding tumorigenesis is one of high genotoxic stress. In order for tumor cells to remain viable not only in their area of primary induction, but also for metastasis and recolonization, a functional DDR needs to maintain a threshold level of genomic integrity within the cell and may also be a prerequisite for tumor cells to survive. Also, because DNA repair mechanisms are modulated in tumor cells to promote survival under genotoxic stress, tumor environments can be more sensitive to inhibition of such DNA repair systems that they have come to be so heavily reliant on. Inhibiting tumor-specific DSB repair pathways, such as those involving RAD52, may function by amplifying DNA damage, overloading the cancer cell with genomic instability to the point where it can no longer thrive [30]. Thus, the manipulation of DNA repair pathways in cancer may play a key role in promoting tumori-genesis and progression by maintaining genomic stability within tumor cells and actually enhancing their survival [53].

### Involvement of the DDR and RAD52 in cell death pathways

Emerging data suggests that inhibition of DNA repair may be inducing cell death through both apoptotic, as well as necrotic means. Apoptosis, the most described form of cell death, is defined as programmed cell death due to environmental cues or intrinsic cell stress [54]. Apoptotic cell death is an energy-dependent method of cell suicide where the content of the cell degrades without rupturing the outer cell membrane or inducing an inflammatory response [55]. Necrosis on the other hand, has up until recently been noted as a solely unregulated form of cell death which occurs due to spontaneous states of trauma within the cell [56]. Recent reports, however, describe a form of cell death by necrosis in which the cell can regulate its own death in response to different forms of cellular damage, including alkylating DNA damage [54].

It has been shown that tumor cells such as follicular lymphoma cells which are found to be resistant to apoptotic-based therapies succumb to cell death when exposed to DNA-alkylating agents and that their death occurs independently of apoptotic effector proteins such as p53, caspases, Bax, and Bak [54, 57]. Along these lines, our own unpublished data shows that depletion of RAD52 in human squamous cell lung carcinoma cell lines enhances phenotypes associated with decreased tumorigenesis and increased cell death. In additions, analysis of sacrificed murine lungs by FACS suggests that knockout appears to increase cell death pathways when treated with the alkylating agent and nitrosourea, NTCU [unpublished data]. Such functional studies continue to support genetic findings by revealing that loss of RAD52 may induce DNA damage and genomic instability within squamous lung tumor cells beyond a threshold of viability. Thus, our data suggests a role for regulated necrosis in combating lung carcinogenesis by depletion of RAD52 and subsequent inhibition of the DNA damage response.

## TARGETING THE DDR AS A MECHANISM IN TUMORIGENESIS

### Inhibiting Rad52 function to thwart tumorigenesis

Although squamous cell lung cancer is responsible for approximately 400,000 deaths each year worldwide, like many diseases, there are no drugs specifically designed to target this particular type of cancer [58]. In accordance, several groups have begun identifying and optimizing small molecule inhibitors against proteins which are believed to enhance tumorigenesis, such as RAD52, to better treat lung cancers and other diseases. Targeting Rad52 has been shown to eliminate cancer stem and progenitor cells, induce high levels of spon-taneous DNA damage and decrease tumorigenesis without detectable effects on normal cells and tissues [30]. Thus, RAD52 represents an attractive, potential therapeutic target and remains high among the list of current inhibitor targets [30, 31, 59, 60].

Two promising techniques for targeting RAD52 have been demonstrated through the use of aptamers which target the binding ability of RAD52 to DNA, as well as small molecule inhibitors targeting RAD52 based on its structure [30, 32, 60]. These RAD52 inhibitors have therapeutic potential on many levels. When combined with mutations in genes that cause hereditary breast and ovarian cancer such as BRCA1, BRCA2, PALB2 and RAD51C, depletion of Rad52 is shown to be synthetically lethal [27]. In the context of cancer, synthetic lethality results when a genetic mutation in a single gene leaves the cell viable, but simultaneous mutations in two genes leads to cell death [61]. So, targeting a gene that is synthetic lethal to a cancer-relevant mutation should kill only cancer cells and spare normal cells. Synthetic lethality, therefore, provides a conceptual framework for the development of cancer-specific cytotoxic agents. This finding increased interest in developing new ways of targeting Rad52 expression and activity. Along these lines, Huang et al. identified 70 presumed inhibitors of RAD52 which were then used to develop small molecule inhibitors of the RAD52 ssDNA annealing activity [32]. Functional results using these inhibitors demonstrated that inhibiting RAD52, in a model of hereditary breast and ovarian cancer, works to suppress growth of BRCA1- and BRCA2-deficient cells, impeding tumorigenesis [32].

While a specific target has been demonstrated in BRCA1/2 deficient cancers, proving that advances in the treatment of primary breast cancer are on the horizon, breast-to-brain metastases remains difficult to treat. Penetration of drugs through the blood-brain barrier (BBB) is a major determinant of therapeutic efficacy against brain metastatic tumor cells [62–64]. Vorinostat, a histone deactylase inhibitor, showed significant uptake into normal brain, and significantly prevented the development of brain metastases of breast cancer in an *in vivo* model [65]. Increased PCNA staining of vorinostat-treated brain metastases *in vivo* suggested a response to DNA damage as PCNA is a processivity factor for DNA polymerases in both DNA replication and DNA repair [65]. The implication of DNA damage as a mechanism of action then lead to further analysis by microarray where Rad52 downregulation was identified and thought to be as a consequence of vorinostat treatment *in vivo*. Single agent inhibitory activity of vorinostat was thus linked to a novel function. As the first preclinical data for the prevention of brain metastasis of triple-negative breast cancer, these observations suggested that induction of DNA double-strand breaks by Vorinostat was associated with the downregulation of the DNA repair gene Rad52 [65]. Thus, it was hypothesized that decreased expression of Rad52 may enhance DNA DSBs by impairing repair and allowing overwhelming DNA damage to accumulate, resulting in decreased viability and a slower rate of tumor cell outgrowth [65].

Although the exact mechanism of RAD52 function in HR in mammalian cells is not yet completely understood, RAD52 has been determined to retain ssDNA annealing activity both *in vitro* and *in vivo*, which it utilizes to function in HR [32, 66]. Researchers in the Skorski group have identified compounds capable of disrupting ssDNA binding by RAD52 and which consequently exerted anti-tumor activity against BRCA-mutated carcinoma cells [60]. This group determined FDA approved drugs or drug-like active compounds which bind to a critical “hotspot” in the RAD52 DNA binding domain 1. One of the compounds, adenosine 5′-monophosphate (A5MP), as well as its mimic 5-aminoimidazole-4-carboxamide ribonucleotide (AICAR) 5′ phosphate (ZMP) inhibited RAD52 activity *in vivo*. As previously suggested, this activity exerted synthetic lethality against BRCA1 and BRCA2–mutated carcinomas. These data shed further light on the use of active inhibitory compounds against RAD52 for the development drug-inducing synthetic lethality to combat tumors [60].

Thus, through either peptide aptamers, small molecule inhibitors, or chemotherapeutic histone deactylase inhibitors, RAD52 inhibition can be used as a prototype for development of novel therapies against hereditary cancers with defective BRCA proteins or can be applied against sporadic cancers in which BRCA1 or BRCA2 are either mutated or downregulated due to mechanisms such as constitutive promoter methylation [67, 68]. With this new technology, researchers have already begun to demonstrate a mechanism of treatment targeting both hereditary breast and ovarian cancers, as well as BCR-ABL1 positive leukemia cells [30, 67]. As discussed above, DNA repair machinery plays a critical role in the induction, progression, and treatment of cancer [69]. Thus, by pharmacological targeting of tumor-specific DNA repair pathways functions in this unique manner, therapy is able to amplify endogenous and drug-induced DNA damage and trigger cell death in cancer cells while sparing both normal cells and heightened toxicity in the body [60].

### CONCLUDING REMARKS

Malignant cells from both advanced solid tumors and hematological cancers already contain a high level of potentially lethal spontaneous DSBs caused by endogenous factors such as ROS, AID, RAG1/1, and upregulated oncogenes [70–74]. Thus, by eliminating key elements in the DNA damage response, pro-vocation of synthetic lethality (i.e., in BRCA deficient cells) or sensitization of otherwise-resistant tumor cells may be possible through generation of an overwhelming level of spontaneous and lethal DNA damage. Remarkably, this method of treatment may even be efficacious without genotoxic treatment, (i.e., effects produced by chemotherapeutics) or with lower doses of treatment.

Furthermore, unlike previous data showing that knockout models of Rad51 are lethal to normal cells both *in vivo* and *in vitro*, this precise method solely effects tumor cells and does not cause damage to normal cells because recombinational repair is mostly active in S-phase of the cell cycle where DNA is being replicated at a high rate in ultra-proliferative tumor cells [75, 76]. Thus, this demonstration of efficacy against tumorigenesis by targeting RAD52 through either its expression or by preventing RAD52 from binding to DNA not only suggests the potential benefit of systematic gene targeting as a cancer therapy, but also shows that there may be a way to couple such gene targeting with a less cytotoxic treatment to combat otherwise resistant tumor cells [30].

Recent advances in genome analysis have identified a number of genetic alterations in cancer that could be therapeutically exploited or used as predictive bio markers for guiding treatment decisions, customizing therapy, and eventually improving patient outcome [53]. Oncogenic data surrounding RAD52 and the DNA damage response elucidate how DNA damage and repair remain at the cornerstone of cancer at every level, modulating the disease from initiation to progression and even throughout treatment. Inhibiting DDR pathways that tumor cells have come to rely on for sustaining viability may work to combat the cancer by amplifying both endogenous and drug-induced DNA damage, thus, triggering cell death in tumor cells while sparing normal cells that do not rely on these pathways to maintain their integrity [30, 77–79]. Thus, by targeting Rad52, the ensuing inhibition of cancer cell-related DNA repair may amplify both endogenous and drug-induced DNA damage, sensitizing tumor cells to cell death [30]. Finally, beyond the scope of induced synthetic lethality in BRCA deficient cells, this Rad52-targeted phenotype may also be proven relevant by genetic and epigenetic deficiencies found in other genes, lending to the importance of prototypic Rad52 inhibitors currently in the pipeline.

**Figure 1:**
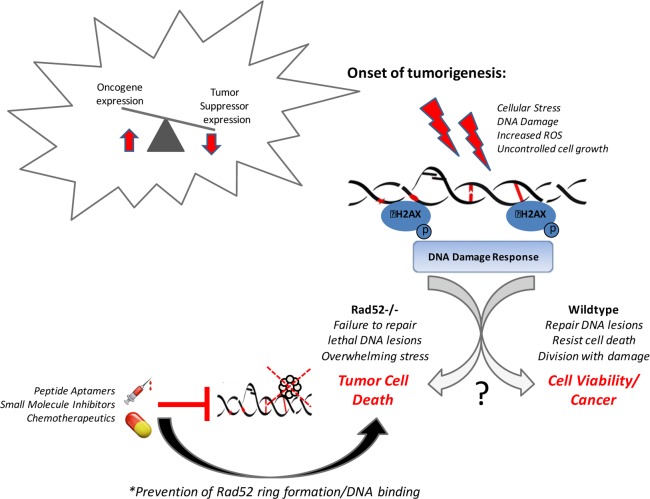
Rad52 activity promotes tumor cell viability

Our recent work [84] suggests that cell death, tumor inhibition, and immunity are enhanced in mouse cells depleted of RAD52. As shown in Figure 1, loss of Rad52 increases genomic instability beyond a manageable threshold and consents the damaged cells to death before they are able to become tumor cells. We have also shown a key role for the complex interplay between the DNA damage response and host immunity in determining risk for LUSC [84]. DNA damage has also long been regarded as one of the primary driving processes of aging contributing to genomic instability and some of the premature aging diseases are the consequence of accumulated DNA damage [85, 86]. Further investigations into the link between mediators of the DDR and tumorigenesis and aging are required to de-lineate the mechanism of this important facet of NSCLC disease progression and premature aging diseases.

## References

[1] Khanna KK, Jackson SP (2001). DNA double-strand breaks: signaling, repair and the cancer connection. Nat Genet.

[2] Boesch P, Weber-Lotfi F, Ibrahim N, Tarasenko V, Cosset A, Paulus F, Lightowlers RN, Dietrich A (2011). DNA repair in organelles: Pathways, organization, regulation, relevance in disease and aging. Biochim Biophys Acta.

[3] Harper JW, Elledge SJ (2007). The DNA damage response: ten years after. Mol Cell.

[4] Lindahl T, Barnes DE (2000). Repair of endogenous DNA damage. Cold Spring Harb Symp Quant Biol.

[5] Shiloh Y (2003). ATM and related protein kinases: safeguarding genome integrity. Nat Rev Cancer.

[6] Roos WP, Kaina B (2013). DNA damage-induced cell death: from specific DNA lesions to the DNA damage response and apoptosis. Cancer Lett.

[7] Hoeijmakers JH (2001). DNA repair mechanisms. Maturitas.

[8] Hoeijmakers JH (2009). DNA damage, aging, and cancer. N Engl J Med.

[9] Shen Z (2011). Genomic instability and cancer: an introduction. J Mol Cell Biol.

[10] Jackson SP, Bartek J (2009). The DNA-damage response in human biology and disease. Nature.

[11] Polo SE, Jackson SP (2011). Dynamics of DNA damage response proteins at DNA breaks: a focus on protein modifications. Genes Dev.

[12] O'Connor MJ (2015). Targeting the DNA Damage Response in Cancer. Mol Cell.

[13] Branzei D, Foiani M (2008). Regulation of DNA repair throughout the cell cycle. Nat Rev Mol Cell Biol.

[14] Nik-Zainal S, Alexandrov LB, Wedge DC, Van Loo P, Greenman CD, Raine K, Jones D, Hinton J, Marshall J, Stebbings LA, Menzies A, Martin S, Leung K, Breast Cancer Working Group of the International Cancer Genome Consortium (2012). Mutational processes molding the genomes of 21 breast cancers. Cell.

[15] Helleday T, Eshtad S, Nik-Zainal S (2014). Mechanisms underlying mutational signatures in human cancers. Nat Rev Genet.

[16] Jackson AL, Loeb LA (2001). The contribution of endogenous sources of DNA damage to the multiple mutations in cancer. Mutat Res.

[17] d'Adda di Fagagna F, Reaper PM, Clay-Farrace L, Fiegler H, Carr P, Von Zglinicki T, Saretzki G, Carter NP, Jackson SP (2003). A DNA damage checkpoint response in telomere-initiated senescence. Nature.

[18] Freund A, Orjalo AV, Desprez PY, Campisi J (2010). Inflam-matory networks during cellular senescence: causes and consequences. Trends Mol Med.

[19] Davis AJ, Chen DJ (2013). DNA double strand break repair via non-homologous end-joining. Transl Cancer Res.

[20] Lieber MR (2010). The mechanism of double-strand DNA break repair by the nonhomologous DNA end-joining pathway. Annu Rev Biochem.

[21] Symington LS (2002). Role of RAD52 epistasis group genes in homologous recombination and double-strand break repair. Microbiol Mol Biol Rev.

[22] Li X, Heyer WD (2008). Homologous recombination in DNA repair and DNA damage tolerance. Cell Res.

[23] Liu J, Heyer WD (2011). Who's who in human recombination: BRCA2 and RAD52. Proc Natl Acad Sci USA.

[24] Game JC, Mortimer RK (1974). A genetic study of x-ray sensitive mutants in yeast. Mutat Res.

[25] Resnick MA (1969). Genetic control of radiation sensitivity in Saccharomyces cerevisiae. Genetics.

[26] Dong Z, Fasullo M (2003). Multiple recombination pathways for sister chromatid exchange in Saccharomyces cerevisiae: role of RAD1 and the RAD52 epistasis group genes. Nucleic Acids Res.

[27] Feng Z, Scott SP, Bussen W, Sharma GG, Guo G, Pandita TK, Powell SN (2011). Rad52 inactivation is synthetically lethal with BRCA2 deficiency. Proc Natl Acad Sci USA.

[28] Liu Y, Maizels N (2000). Coordinated response of mammalian Rad51 and Rad52 to DNA damage. EMBO Rep.

[29] Lisby M, Rothstein R, Mortensen UH (2001). Rad52 forms DNA repair and recombination centers during S phase. Proc Natl Acad Sci USA.

[30] Cramer-Morales K, Nieborowska-Skorska M, Scheibner K, Padget M, Irvine DA, Sliwinski T, Haas K, Lee J, Geng H, Roy D, Slupianek A, Rassool FV, Wasik MA (2013). Personalized synthetic lethality induced by targeting RAD52 in leukemias identified by gene mutation and expression profile. Blood.

[31] Lok BH, Carley AC, Tchang B, Powell SN (2013). RAD52 inactivation is synthetically lethal with deficiencies in BRCA1 and PALB2 in addition to BRCA2 through RAD51-mediated homologous recombination. Oncogene.

[32] Huang F, Goyal N, Sullivan K, Hanamshet K, Patel M, Mazina OM, Wang CX, An WF, Spoonamore J, Metkar S, Emmitte KA, Cocklin S, Skorski T, Mazin AV (2016). Targeting BRCA1- and BRCA2-deficient cells with RAD52 small molecule inhibitors. Nucleic Acids Res.

[33] Treuner K, Helton R, Barlow C (2004). Loss of Rad52 partially rescues tumorigenesis and T-cell maturation in Atm-deficient mice. Oncogene.

[34] Lieberman R, Xiong D, James M, Han Y, Amos CI, Wang L, You M (2016). Functional characterization of RAD52 as a lung cancer susceptibility gene in the 12p13.33 locus. Mol Carcinog.

[35] Meyerson M, Gabriel S, Getz G (2010). Advances in understanding cancer genomes through second-generation sequencing. Nat Rev Genet.

[36] Govindan R, Ding L, Griffith M, Subramanian J, Dees ND, Kanchi KL, Maher CA, Fulton R, Fulton L, Wallis J, Chen K, Walker J, McDonald S (2012). Genomic landscape of non-small cell lung cancer in smokers and never-smokers. Cell.

[37] Tang S, Pan Y, Wang Y, Hu L, Cao S, Chu M, Dai J, Shu Y, Xu L, Chen J, Jin G, Hu Z, Ma H, Shen H (2015). Genome-wide association study of survival in early-stage non-small cell lung cancer. Ann Surg Oncol.

[38] Cao S, Wang S, Ma H, Tang S, Sun C, Dai J, Wang C, Shu Y, Xu L, Yin R, Song X, Chen H, Han B (2016). Genome-wide association study of myelosuppression in non-small-cell lung cancer patients with platinum-based chemotherapy. Pharmacogenomics J.

[39] Shi J, Chatterjee N, Rotunno M, Wang Y, Pesatori AC, Consonni D, Li P, Wheeler W, Broderick P, Henrion M, Eisen T, Wang Z, Chen W (2012). Inherited variation at chromosome 12p13.33, including RAD52, influences the risk of squamous cell lung carcinoma. Cancer Discov.

[40] de Souza-Pinto NC, Maynard S, Hashiguchi K, Hu J, Muftuoglu M, Bohr VA (2009). The recombination protein RAD52 cooperates with the excision repair protein OGG1 for the repair of oxidative lesions in mammalian cells. Mol Cell Biol.

[41] Kenfield SA, Wei EK, Stampfer MJ, Rosner BA, Colditz GA (2008). Comparison of aspects of smoking among the four histological types of lung cancer. Tob Control.

[42] Naccarati A, Rosa F, Barone E, Jiraskova K, Di Gaetano C, Novotny J, Levy M, Vodickova L, Gemignani F, Buchler T, Landi S, Vodicka P, Pardini B (2016). Double-strand break repair and colorectal cancer: gene variants within 3′ UTRs and microRNAs binding as modulators of cancer risk and clinical outcome. Oncotarget.

[43] Shivapurkar N, Mikhail S, Navarro R, Bai W, Marshall J, Hwang J, Pishvaian M, Wellstein A, He AR (2013). Decrease in blood miR-296 predicts chemotherapy resistance and poor clinical outcome in patients receiving systemic chemotherapy for metastatic colon cancer. Int J Colorectal Dis.

[44] van Jaarsveld MT, Wouters MD, Boersma AW, Smid M, van Ijcken WF, Mathijssen RH, Hoeijmakers JH, Martens JW, van Laere S, Wiemer EA, Pothof J (2014). DNA damage responsive microRNAs misexpressed in human cancer modulate therapy sensitivity. Mol Oncol.

[45] Iorio MV, Croce CM (2012). Causes and consequences of microRNA dysregulation. Cancer J.

[46] Shi TY, Yang G, Tu XY, Yang JM, Qian J, Wu XH, Zhou XY, Cheng X, Wei Q (2012). RAD52 variants predict platinum resistance and prognosis of cervical cancer. PLoS One.

[47] Lissoni AA, Colombo N, Pellegrino A, Parma G, Zola P, Katsaros D, Chiari S, Buda A, Landoni F, Peiretti M, Dell'anna T, Fruscio R, Signorelli M (2009). A phase II, randomized trial of neo-adjuvant chemotherapy comparing a three-drug combination of paclitaxel, ifosfamide, and cisplatin (TIP) versus paclitaxel and cisplatin (TP) followed by radical surgery in patients with locally advanced squamous cell cervical carcinoma: the Snap-02 Italian Collaborative Study. Ann Oncol.

[48] Li F, Sun X, Sun N, Qin S, Cheng H, Feng J, Chen B, Cheng L, Lu Z, Ji J, Zhou Y (2010). Association between polymorphisms of ERCC1 and XPD and clinical response to platinum-based chemotherapy in advanced non-small cell lung cancer. Am J Clin Oncol.

[49] Hironaka K, Factor VM, Calvisi DF, Conner EA, Thorgeirsson SS (2003). Dysregulation of DNA repair pathways in a transforming growth factor alpha/c-myc transgenic mouse model of accelerated hepatocarcinogenesis. Lab Invest.

[50] Barlow C, Hirotsune S, Paylor R, Liyanage M, Eckhaus M, Collins F, Shiloh Y, Crawley JN, Ried T, Tagle D, Wynshaw-Boris A (1996). Atm-deficient mice: a paradigm of ataxia telangiectasia. Cell.

[51] Alcalay M, Meani N, Gelmetti V, Fantozzi A, Fagioli M, Orleth A, Riganelli D, Sebastiani C, Cappelli E, Casciari C, Sciurpi MT, Mariano AR, Minardi SP (2003). Acute myeloid leukemia fusion proteins deregulate genes involved in stem cell maintenance and DNA repair. J Clin Invest.

[52] Shaheen M, Allen C, Nickoloff JA, Hromas R (2011). Synthetic lethality: exploiting the addiction of cancer to DNA repair. Blood.

[53] Curtin NJ (2012). DNA repair dysregulation from cancer driver to therapeutic target. Nat Rev Cancer.

[54] Zong WX, Ditsworth D, Bauer DE, Wang ZQ, Thompson CB (2004). Alkylating DNA damage stimulates a regulated form of necrotic cell death. Genes Dev.

[55] Wang X (2001). The expanding role of mitochondria in apoptosis. Genes Dev.

[56] Kanduc D, Mittelman A, Serpico R, Sinigaglia E, Sinha AA, Natale C, Santacroce R, Di Corcia MG, Lucchese A, Dini L, Pani P, Santacroce S, Simone S (2002). Cell death: apoptosis versus necrosis (review). Int J Oncol.

[57] Lister TA (1991). The management of follicular lymphoma. Ann Oncol.

[58] Hammerman PS, Lawrence MS, Voet D, Jing R, Cibulskis K, Sivachenko A, Stojanov P, McKenna A, Lander ES, Gabriel S, Getz G, Sougnez C, Imielinski M, Cancer Genome Atlas Research Network (2012). Comprehensive genomic characterization of squamous cell lung cancers. Nature.

[59] Huang F, Goyal N, Sullivan K, Hanamshet K, Patel M, Mazina OM, Wang CX, An WF, Spoonamore J, Metkar S, Emmitte KA, Cocklin S, Skorski T, Mazin AV (2016). Targeting BRCA1- and BRCA2-deficient cells with RAD52 small molecule inhibitors. Nucleic Acids Res.

[60] Sullivan K, Cramer-Morales K, McElroy DL, Ostrov DA, Haas K, Childers W, Hromas R, Skorski T (2016). Identification of a Small Molecule Inhibitor of RAD52 by Structure-Based Selection. PLoS One.

[61] Kaelin WG (2005). The concept of synthetic lethality in the context of anticancer therapy. Nat Rev Cancer.

[62] Lin NU, Bellon JR, Winer EP (2004). CNS metastases in breast cancer. J Clin Oncol.

[63] Weil RJ, Palmieri DC, Bronder JL, Stark AM, Steeg PS (2005). Breast cancer metastasis to the central nervous system. Am J Pathol.

[64] Bart J, Groen HJ, Hendrikse NH, van der Graaf WT, Vaalburg W, de Vries EG (2000). The blood-brain barrier and oncology: new insights into function and modulation. Cancer Treat Rev.

[65] Palmieri D, Lockman PR, Thomas FC, Hua E, Herring J, Hargrave E, Johnson M, Flores N, Qian Y, Vega-Valle E, Taskar KS, Rudraraju V, Mittapalli RK (2009). Vorinostat inhibits brain metastatic colonization in a model of triple-negative breast cancer and induces DNA double-strand breaks. Clin Cancer Res.

[66] Stark JM, Pierce AJ, Oh J, Pastink A, Jasin M (2004). Genetic steps of mammalian homologous repair with distinct mutagenic consequences. Mol Cell Biol.

[67] Hansmann T, Pliushch G, Leubner M, Kroll P, Endt D, Gehrig A, Preisler-Adams S, Wieacker P, Haaf T (2012). Constitutive promoter methylation of BRCA1 and RAD51C in patients with familial ovarian cancer and early-onset sporadic breast cancer. Hum Mol Genet.

[68] Esteller M (2008). Epigenetics in cancer. N Engl J Med.

[69] Helleday T, Petermann E, Lundin C, Hodgson B, Sharma RA (2008). DNA repair pathways as targets for cancer therapy. Nat Rev Cancer.

[70] Sallmyr A, Fan J, Rassool FV (2008). Genomic instability in myeloid malignancies: increased reactive oxygen species (ROS), DNA double strand breaks (DSBs) and error-prone repair. Cancer Lett.

[71] Callén E, Jankovic M, Difilippantonio S, Daniel JA, Chen HT, Celeste A, Pellegrini M, McBride K, Wangsa D, Bredemeyer AL, Sleckman BP, Ried T, Nussenzweig M, Nussenzweig A (2007). ATM prevents the persistence and propagation of chromosome breaks in lymphocytes. Cell.

[72] Hasham MG, Donghia NM, Coffey E, Maynard J, Snow KJ, Ames J, Wilpan RY, He Y, King BL, Mills KD (2010). Widespread genomic breaks generated by activation-induced cytidine deaminase are prevented by homologous recombination. Nat Immunol.

[73] Nieborowska-Skorska M, Kopinski PK, Ray R, Hoser G, Ngaba D, Flis S, Cramer K, Reddy MM, Koptyra M, Penserga T, Glodkowska-Mrowka E, Bolton E, Holyoake TL (2012). Rac2-MRC-cIII-generated ROS cause genomic instability in chronic myeloid leukemia stem cells and primitive progenitors. Blood.

[74] Seo J, Kim SC, Lee HS, Kim JK, Shon HJ, Salleh NL, Desai KV, Lee JH, Kang ES, Kim JS, Choi JK (2012). Genome-wide profiles of H2AX and γ-H2AX differentiate endogenous and exogenous DNA damage hotspots in human cells. Nucleic Acids Res.

[75] Karanam K, Kafri R, Loewer A, Lahav G (2012). Quantitative live cell imaging reveals a gradual shift between DNA repair mechanisms and a maximal use of HR in mid S phase. Mol Cell.

[76] Tsuzuki T, Fujii Y, Sakumi K, Tominaga Y, Nakao K, Sekiguchi M, Matsushiro A, Yoshimura Y, Morita T (1996). Targeted disruption of the Rad51 gene leads to lethality in embryonic mice. Proc Natl Acad Sci USA.

[77] Negrini S, Gorgoulis VG, Halazonetis TD (2010). Genomic instability--an evolving hallmark of cancer. Nat Rev Mol Cell Biol.

[78] Hanahan D, Weinberg RA (2011). Hallmarks of cancer: the next generation. Cell.

[79] Ciccia A, Elledge SJ (2010). The DNA damage response: making it safe to play with knives. Mol Cell.

[80] Liang Z, Ahn J, Guo D, Votaw JR, Shim H (2013). MicroRNA-302 replacement therapy sensitizes breast cancer cells to ionizing radiation. Pharm Res.

[81] Li Z, Guo Y, Zhou L, Ge Y, Wei L, Li L, Zhou C, Wei J, Yuan Q, Li J, Yang M (2015). Association of a functional RAD52 genetic variant locating in a miRNA binding site with risk of HBV-related hepatocellular carcinoma. Mol Carcinog.

[82] Pan HC, Lai DW, Lan KH, Shen CC, Wu SM, Chiu CS, Wang KB, Sheu ML (2013). Honokiol thwarts gastric tumor growth and peritoneal dissemination by inhibiting Tpl2 in an orthotopic model. Carcinogenesis.

[83] Chandramouly G, McDevitt S, Sullivan K, Kent T, Luz A, Glickman JF, Andrake M, Skorski T, Pomerantz RT (2015). Small-Molecule Disruption of RAD52 Rings as a Mechanism for Precision Medicine in BRCA-Deficient Cancers. Chem Biol.

[84] Lieberman R, Pan J, Zhang Q, You M (2017). Rad52 deficiency decreases development of lung squamous cell carcinomas by enhancing immuno-surveillance. Oncotarget.

[85] He M, Lin Y, Tang Y, Liu Y, Zhou W, Li C, Sun G, Guo M (2016). miR-638 suppresses DNA damage repair by targeting SMC1A expression in terminally differentiated cells. Aging (Albany NY).

[86] Nicolai S, Rossi A, Di Daniele N, Melino G, Annicchiarico-Petruzzelli M, Raschellà G (2015). DNA repair and aging: the impact of the p53 family. Aging (Albany NY).

